# Explaining the age‐moderation effects in the relation between immediate benefits and physical activity: A mediated moderation analysis

**DOI:** 10.1111/bjhp.70006

**Published:** 2025-07-14

**Authors:** Kin‐Kit Li, Wanying Zhao, Cyrus Lap Kwan Leung

**Affiliations:** ^1^ Department of Social and Behavioural Sciences City University of Hong Kong Hong Kong SAR China; ^2^ The Jockey Club School of Public Health and Primary Care The Chinese University of Hong Kong Hong Kong SAR China

**Keywords:** future‐time perspective, Hong Kong, immediate benefits, mediated moderation, older adults, physical activity

## Abstract

**Background:**

Older adults are the least physically active age segment. Understanding age‐related determinants of physical activity remains a priority. While distal benefits of physical activity (PA) are well reported, immediate benefits can also enhance PA. Older adults, perceiving future time as more limited, may find immediate benefits more motivating. In addition, older adults are more health‐conscious and may find PA benefits consistent with their belief system.

**Aims:**

This study examined whether increased age was associated with a shortened future‐time perspective and increased health consciousness, which strengthened the association between immediate benefits and subsequent PA.

**Methods:**

In a prospective survey, 241 older and 180 younger adults reported their perceived importance of immediate PA benefits, future‐time perspective and health consciousness at baseline and reported their past 7‐day PA at a one‐week follow‐up.

**Results:**

The total mediated moderation effect was significant, *b* = .05 (95% bias‐corrected CI: .01, .10). Specifically, the mediated moderation effects of future‐time perspective was significant, *b* = .02 (.002, .06) but that of health consciousness was not, *b* = .03 (−.01, .06). Surprisingly, the direct age moderation was significant and negative, *b* = −.16 (−.27, −.05), indicating the relation between immediate benefits and PA was stronger among younger adults.

**Discussion:**

As expected, older adults perceived future time as more limited, and hence, immediate benefits were more predictive of PA.

**Conclusion:**

The findings support the time perspective concordance hypothesis and suggest that younger adults may find immediate benefits motivating for very different reasons that require further investigation.

## INTRODUCTION

Adults aged 65 or above in the total population were projected to increase from 10% in 2022 to 16% in 2050 globally, and the percentage was projected to reach 27% in the Europe and Northern America region (United Nations Department for Economic and Social Affairs, 2023). The demands for healthcare services increase inevitably with population aging (Strunk et al., 2006). Adopting a healthy lifestyle, such as participating in regular physical activity (PA), can help prevent various health conditions such as hypertension, cardiovascular disease, cancer, type 2 diabetes, depression and anxiety and cognitive decline (Warburton et al., 2006), which may subsequently alleviate the increasing age‐related healthcare costs (Leigh et al., 2005). However, 28% of adults do not engage in PA at the recommended level for the benefits, and the prevalence of inactivity was more common in high‐income countries and increased with age (World Health Organization, 2022). Therefore, understanding the age‐related determinants of PA remains a research priority.

While the long‐term physical benefits of PA are often the main focus in epidemiological studies (e.g., Reiner et al., 2013), a recent scoping review showed that short‐term social and mental health outcomes were often included in the message content across different age groups in those studies promoting PA through messaging (Williamson et al., 2020). However, the extent to which these short‐term benefits can effectively promote PA across different age groups is unknown. This study examined whether two age‐related constructs, future‐time perspective (FTP) and health consciousness, could explain any age differences in the relation between immediate benefits and PA.

### Future‐time perspective and physical activity

FTP is defined broadly as the motivational mechanism that links immediate actions with future consequences (e.g., Lens et al., 2012; Wininger & Desena, 2012). That is, individuals who are more future‐oriented can identify and value future benefits that will drive the actions despite possible immediate costs. Although they appear in different conceptualizations, including disposition traits (Zimbardo & Boyd, 1999), the expandability of future time (Carstensen & Lang, 1996) and consideration for future consequences (Strathman et al., 1994), all concepts of future‐time perspectives share important implications for the regulation of health behaviours (e.g., Hall et al., 2015; Löckenhoff & Carstensen, 2007).

Many health outcomes of PA participation are long‐term (e.g., improvement in fitness, prevention of chronic diseases). It is believed that the ability to value the delayed rewards of PA over the immediate rewards of competing activities is important for PA participation (e.g., Hall et al., 2015). Therefore, the ability to recognize the distal rewards (i.e., a strong FTP) and to act upon them should be one important strategy to promote PA. Expectedly, a FTP has been shown to be associated with increased PA (e.g., Adams, 2009; Guthrie et al., 2014; Stahl & Patrick, 2012).

### The importance of immediate rewards

There has been growing interest in examining the importance of immediate rewards for health‐related behaviours, including PA. The cumulating evidence suggests that immediate rewards are as important as, if not more than, the delayed rewards for motivating PA (e.g., Daugherty & Brase, 2010; Evans et al., 2019; Li, 2013; Woolley & Fishbach, 2017).

When using a multidimensional approach to measure time perspective, a higher level in both future perspective and present‐hedonistic perspective predicted more exercise in a cross‐sectional study among college students (Daugherty & Brase, 2010), indicating pleasant immediate outcomes may be effective in promoting PA. Using a latent profile analysis, Li (2013) found four groups of middle‐aged and older adults according to their evaluation of the importance and immediacy of outcome expectancy of PA. Outcome expectancy refers to the belief that specific outcomes are contingent on the performance of certain behaviours. Importance emphasizes the significance of these outcomes, while immediacy denotes how soon the outcomes can be actualized by performing the behaviours. The group that considered PA outcomes as important and immediate was more physically active than the other three groups (i.e., low importance and immediacy, moderate importance and immediacy and high importance and low immediacy). However, this group of individuals only constituted 17% of the sample. In a retrospective study, Woolley and Fishbach (2017) showed that immediate rewards, but not delayed rewards, were predictive of PA in the past 3 months. In a cross‐sectional study, Evans et al. (2019) showed that both the ranked benefits of immediate and delayed rewards predicted self‐reported moderate‐intensity PA, whereas only the ranked immediate benefits predicted vigorous‐intensity PA.

### Age differences in the impact of immediate benefits

While direct evidence for an age moderation on the relationship between the importance of immediate benefits and PA is not readily available in the literature, some previous findings suggest age differences in the preferences for immediate rewards and reasons for engaging in PA, providing indirect support for this possibility. Older and younger adults may be motivated by immediate benefits differently, as shown in delay discounting experiments where participants choose between a smaller immediate reward and a larger delayed reward. However, the findings have been mixed. Some studies have shown that children, adolescents and college students preferred monetary rewards with shorter delays compared to middle‐aged and older adults, suggesting impulsivity may decrease with age (Green et al., 1994; Whelan & McHugh, 2009). Yet, age trends may vary with the characteristics of the competing rewards. For instance, older adults preferred short delays when choices had similar subjective values, implying better working memory and, hence, more deliberate consideration among younger adults (Green et al., 1994). Additionally, older adults were more likely than younger adults to choose immediate rewards when the rewards involved positive social interaction or health improvement, a trend not observed with monetary rewards (Seaman et al., 2016), suggesting context‐specific effects of immediate benefits.

Some studies examining the reasons for engaging in PA may also support an age moderation, although the evidence may lack explicit temporal dimensions or direct age comparisons. For instance, studies have shown that some immediate rewards of PA, such as improved mood and increased energy (Evans et al., 2019; Steltenpohl et al., 2019), are more salient and motivating than distal outcomes in older adults. PA also provides opportunities to spend time with friends and family or make new connections (Campbell et al., 2001; Steltenpohl et al., 2019), which serves as another key immediate benefit motivating older adults in PA engagement (Carstensen et al., 2003; Steltenpohl et al., 2019). The findings align with those on delay discounting, suggesting that older adults prefer health and social rewards with shorter delays (Seaman et al., 2016). Conversely, younger adults are more motivated by self‐focused or instrumental goals, such as achieving personal fitness and competing with peers (Steltenpohl et al., 2019), which may involve planning and future‐oriented outcomes. A meta‐synthesis of 14 studies on older adults' PA experiences found immediate benefits, like fun and quality time in social engagement, were valued more than long‐term health outcomes (Devereux‐Fitzgerald et al., 2016). The potential age differences in the impact of immediate PA benefits on individuals' engagement in PA provide a basis for exploring age‐related mechanisms.

### Age‐related mechanisms: Future‐time perspective and health consciousness

The extent to which immediate benefits of PA may influence PA behaviours may depend on how much the beliefs about immediate PA benefits are consistent with an individual's belief system. FTP and health consciousness are two age‐related behavioural tendencies that reflect our belief systems and may moderate the impact of the immediate benefits of PA.

Based on the evidence from cognitive consistency theories (Gawronski & Brannon, 2019), as developed from Festinger's (1957) cognitive dissonance theory, inconsistent beliefs may hinder information processing and subsequent context‐appropriate behaviours. From an epistemic point of view, encountering inconsistent cognitions may be a signal for an error in the belief system, which may require belief updating (Gawronski, 2012). The identification of inconsistency may also create negative affective responses (Noordewier et al., 2016). From a pragmatic point of view, the inconsistency may also produce different or even conflicting courses of action, which may impede context‐appropriate behavioural actions (Harmon‐Jones et al., 2009).

Older adults perceive future time to be more restrictive (e.g., Li, 2019; Yeung et al., 2012) and that time passes more quickly than for younger adults (John & Lang, 2015). As future time becomes more restrictive, the striving for long‐term goals may become unrealistic and may cause dissatisfaction among older adults (Wrosch et al., 2013). Therefore, a more limited FTP can be adaptive for older adults. According to socioemotional selectivity theory (SST; Carstensen et al., 2003), individuals prefer socially meaningful goals as their perception of future time is limited. On the other hand, goals that can optimize future outcomes (e.g., instrumental and knowledge‐related goals) are preferred when future time is perceived as more expansive (Lang & Carstensen, 2002). Although emotionally adaptive, a shortened FTP may be counterproductive to the investment in physical health for long‐term benefits, such as participation in PA, healthy eating and regular body checks (Ziegelmann et al., 2006). While FTP studies often use broad categorizations in comparing the young‐old (e.g., 60+ as ‘older’; Netz & Raviv, 2004; Steltenpohl et al., 2019), SST suggests this motivational shift in making meaningful goals is gradual, influenced by individuals' perceptions of time and health conditions rather than chronological age alone.

Therefore, as people age, fewer resources may be invested in future‐oriented behaviours, including PA. Consistent with the cognitive consistency theories (Gawronski & Brannon, 2019), the time perspective concordance hypothesis (Li et al., 2021; Zimbardo & Boyd, 1999) suggests that more preferable outcomes are expected when the temporal orientation of the situational demands (e.g., receiving PA promotion messages focusing on future benefits) matches with one's dispositional time perspective. In Li et al.' study, children reported their time perspectives and were randomly assigned to draw either a future self or a present self. Those assigned to draw a picture consistent with their own time perspective showed better performance subsequently in a reading comprehension task than those assigned to the inconsistent drawing. Among people on a university campus, promotion messages framed consistently with one's temporal orientation were found more effective to enhance intention to use sunscreen (Orbell & Kyriakaki, 2008). Although no direct empirical evidence was found among older adults, it is suggested that those who possess restrictive FTP prefer health‐promoting behaviours that bring immediate benefits to living (e.g., reduced stress, time spent with friends) than those emphasizing disease avoidance, a long‐term benefit that cannot be revealed immediately after the behaviours (Hamilton et al., 2003; Zimbardo & Boyd, 1999). Hence, we hypothesized that increased age would be related to more restrictive FTP, and more restrictive FTP should enhance the association between the importance of immediate benefits and PA.

Another relevant age‐related mechanism is that older adults are more conscious about their health conditions (Becker & Arnold, 2004; Hooker, 1992), and such consciousness comprises being aware of one's health, taking responsibility for their own health and being motivated to take part in health‐related behaviours (Hong, 2009). Unlike FTP or the importance of immediate PA benefits, health consciousness does not appear to have a definitive temporal orientation. While being aware of one's health indicates a present orientation, being motivated to conduct health‐related behaviours does not have a definitive temporal frame. As a trait, health consciousness is considered consistent with the importance of the immediate PA benefits, given that PA benefits are predominately portrayed as health‐related, physically and mentally (Bull et al., 2020). As proposed above, a heightened cognitive consistency should benefit the engagement of PA. Therefore, we hypothesized that an increased age would be related to a stronger health consciousness, which would strengthen the association between the importance of immediate benefits and PA.

**FIGURE 1:**
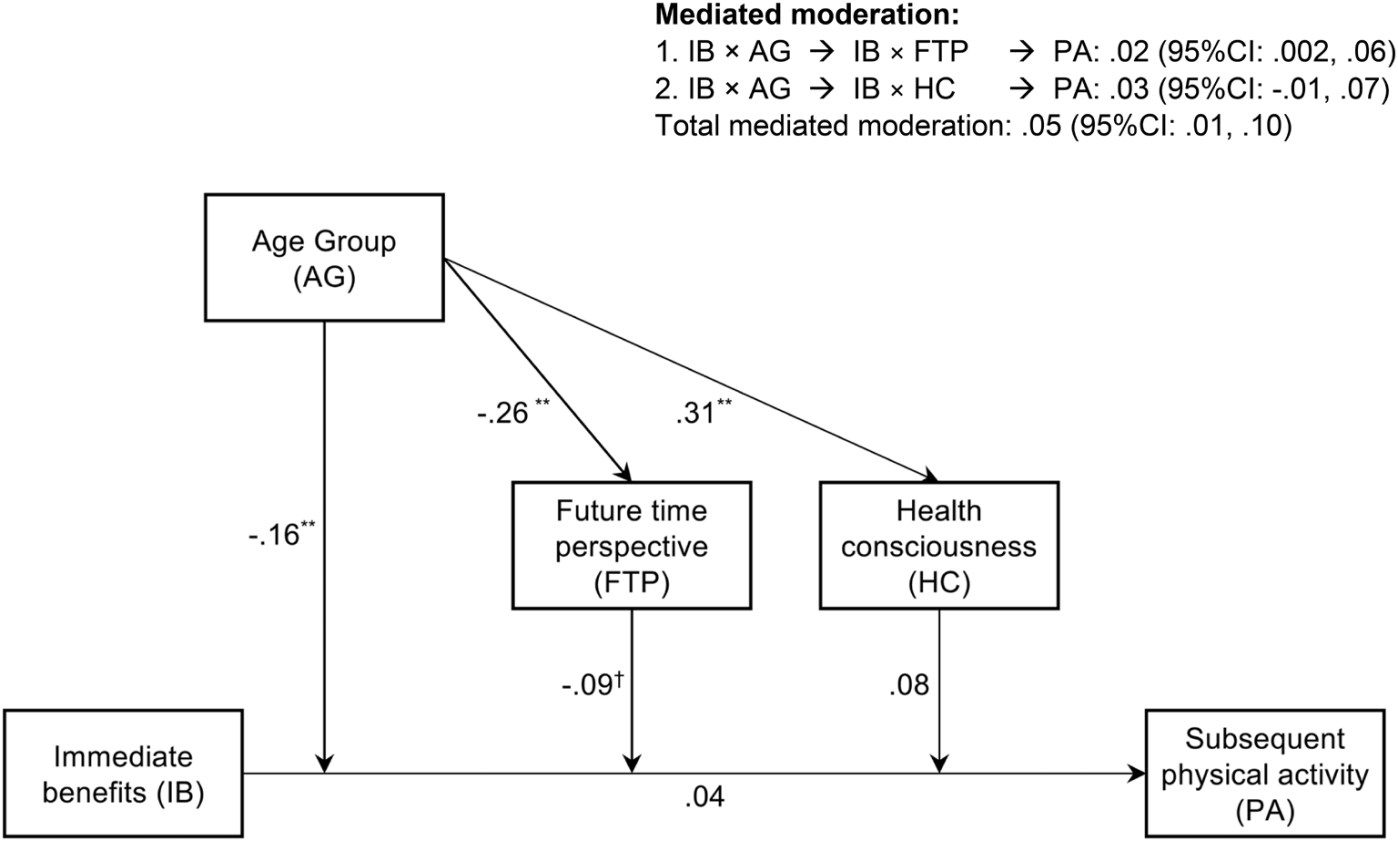
The conceptual mediated moderation model with two mediators (with results).

This study aimed to examine the effects of two age‐related mechanisms, namely FTP and health consciousness, which were suggested to moderate the association between the immediate benefits of PA and PA behaviours among younger and older adults. The proposed relationships are illustrated in Figure 1. Specifically, we would like to test the following hypotheses: (1) Increased age would be related to a more restrictive FTP; (2) A more restrictive FTP should enhance the association between the importance of immediate benefits and PA; (3) Increased age would be related to a stronger health consciousness; (4) A stronger health consciousness would strengthen the association between the importance of immediate benefits and PA.

## METHODS

### Participants

Younger (aged 18–35) and older adults (aged 60 or above) who did not meet the PA recommendation (150 min of moderate‐to‐vigorous intensity PA) and were able to read Chinese were eligible for this study. Individuals who had any physical conditions that prohibited them from participating in PA were excluded. The age ranges and inclusion/exclusion criteria were consistent with previous aging and PA studies (e.g., Li et al., 2014). The estimated total sample size was 342 for a power of .90 given a partial *R* ^2^ of .03 and the number of predictors at 5 at an alpha level of .05 for a single regression coefficient in a linear multiple regression (Faul et al., 2009).

### Procedures

For the recruitment of younger adults, a convenient sampling strategy was used. They were recruited mainly in universities through university email announcements, posters and a participant pool in a psychology course (i.e., students participate in the study for course credits). For older adults, a similar convenient sampling approach was employed. They were recruited in community centres and universities via posters and staff referrals. Participation was voluntary. Students from the participant pool had the option to do an alternative assignment if they chose not to participate in any psychological studies.

Interested individuals had to understand the details of the study and consent to take part. In the first online survey, the participants reported on their FTP, health consciousness and the importance of the immediate benefits of PA. They also provided their demographic and health‐related information and reported their PA behaviour in the past 7 days. One week after the completion of the survey, the participants were asked to report again their PA participation in the past 7 days in the second online survey. Other than the students in the participant pool, the participants received HK$50 cash or equivalent cash coupons as a token of their participation. The study was approved by the Human Subjects Ethics Sub‐Committee of the first author's institution (Reference number: 2‐91‐202,003‐02).

### Instruments

The importance of the immediate benefits of PA underscores the extent to which immediate benefits are personally significant in motivating individuals to engage in PA. In contrast to outcome expectancy, it highlights the personal significance and relevancy but emphasizes less on outcome contingency. It was measured by a single item adapted from a ranking scale in Evans et al.'s (2019) study. Instead of ranking, a 7‐point rating scale format (*not at all important* to *very important*) is adapted based on Woolley and Fishbach's (2017) study. The item was ‘How important is it that you participate in PA for its short‐term benefits (fun, improved mood/energy, reduced stress, etc.)?’ As immediate benefits is a relatively unambiguous construct with a narrower scope among the constructs we measured, that could benefit from the administration time saved in using a single‐item measure (Allen et al., 2022; Wanous et al., 1997). The Chinese version of the International Physical Activity Questionnaire (Macfarlane et al., 2007) was administered to measure the frequency and duration of vigorous‐intensity PA, moderate‐intensity PA, walking and sitting over the past 7 days. Macfarlane et al. have shown that the composite scores were in reasonable agreement with the accelerometer data. The Future‐Time Perspective Scale (Carstensen & Lang, 1996) was used to measure FTP. The scale has been well‐validated and demonstrated good reliability (Lang & Carstensen, 2002). The participants indicated the degree to which they agreed with each of the 10 items on a 7‐point scale (*strongly disagree* to *strongly agree*). Sample items include ‘Many opportunities await me in the future’ and ‘I have the sense that time is running out’. Health consciousness was measured by an 11‐item scale on a 5‐point scale (*strongly disagree* to *strongly agree*) developed by Hong (2009), with good reliability and validity. Sample items included ‘My health depends on how well I take care of myself’ and ‘I notice how I feel physically as I go through the day’.

### Analysis

**FIGURE 2:**
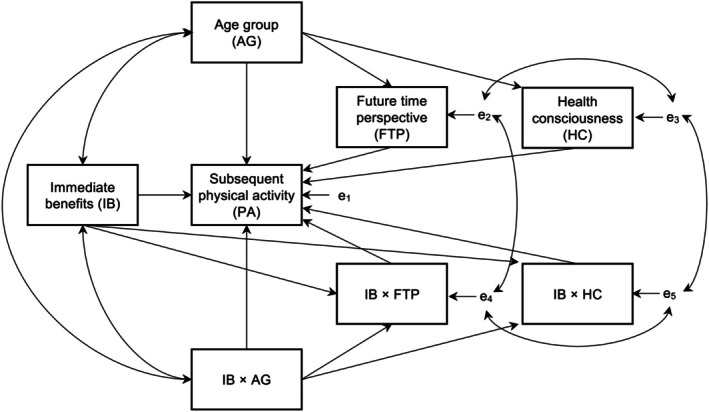
The statistical mediated moderation model.

Descriptive statistics were reported by age groups. PA was scored in the unit of metabolic equivalent task minutes per week (MET‐minute/week), which represented the amount of energy used in PA. The MET‐min/week scores were squared‐root transformed to yield PA scores to enhance normality. We examined the hypothesis by conducting the mediated moderation models suggested by Kwan and Chan (2018), with 10,000 bootstrap samples, using M*plus* (Muthén & Muthén, 1998–2017). We used some abbreviations specifically in this section to show the model specification more clearly. In the model, PA was predicted by immediate benefits (IB), age group (AG), FTP, health consciousness (HC) and the three interactions (i.e., IB × AG, IB × FTP, IB × HC). FTP and health consciousness were predicted by age group. IB × FTP and IB × HC were predicted by IB and IB × AG. The coefficient between AG and FTP and the coefficient between IB × AG and IB × FTP were constrained to be equal. Likewise, the coefficient between AG and HC and the coefficient between IB × AG and IB × HC were constrained to be equal. For model specification, we correlated the variances between IB and AG, between IB and IB × AG and between AG and IB × AG. In addition, we also correlated the residual variances between FTP and IB × FTP and between HC and IB × HC. Finally, we correlated the residual variances between FTP and HC and between IB × FTP and IB × HC. The statistically mediated moderation model is illustrated in Figure 2. Statistical significance was indicated by not containing zero in the 95% bias‐corrected bootstrap confidence interval. Simple slope analyses were conducted to interpret the moderation effects, if any. The participants provided complete information on the surveys as the items were set as mandatory, requiring responses before proceeding to the next page. Missing data due to attrition at the follow‐up was addressed by full information maximum likelihood, which was applied in estimating the mediated moderation model.

We also conducted sensitivity analyses to see how including the covariates might affect the results. In the first sensitivity analysis, gender, body mass index (kg/m^2^), presence of any chronic conditions, perceived health (ranged 1–5) and education level (ranged 1–7) were included in the mediated moderation model as covariates. In the second sensitivity analysis, we added baseline PA in the model. Exogenous variables were allowed to correlate in the models.

## RESULTS

We received 742 and 445 attempts in total in the first and second surveys, respectively, and 467 and 401 included complete responses. Those who provided incomplete responses missed, on average, 84% of the questions and reported very little demographic information, making a meaningful comparison between the completers and non‐completers difficult. A total of 46 participants were excluded because they either failed the attention‐check items or took far less time than average to complete the surveys. The final sample for data analysis comprised 421 participants (241 older, 180 younger) in the first survey and 355 participants (194 older, 161 younger) in the second survey.

An attrition analysis showed that participants who completed the second survey (*n* = 355) were younger than those who dropped out (*n* = 66), with 89% of the younger group and 80% of the older group completing the second survey. They were also more educated, with an average education level of 4.58 (*SD* = 1.33) out of 7, than those who dropped out, *M* = 4.11 (*SD* = 1.42). In addition, they considered immediate benefits less important, scoring 5.54 (*SD* = 1.36) out of 7, than those who dropped out, *M* = 5.92 (*SD* = 1.17). When regressing the demographic factors and studied variables on the completion status of the second survey in a logistic regression, the model was non‐significant, χ^2^ (10) = 17.87, *p* = .06, and none of the regression coefficients were significant. The pseudo‐*R* ^2^ was 4.89%.

In the final sample, 241 adults were aged 60 or above (*M* = 68.27, *SD* = 5.4), and 180 were younger, aged 18 to 35 (*M* = 20.29, *SD* = 2.0). Over 60% of the participants were women. All the participants could participate in PA. Older adults had slightly lower education background and were significantly shorter, *t*(418) = −6.82, *p* < .001. Older adults reported significantly higher weight, *t*(404) = 2.09, *p* = .04, higher BMI, *t*(402) = 7.89, *p* < .001 and better perceived health conditions than younger adults. Older adults were more present‐oriented (*M* = 4.09, *SD* = 1.23) than younger adults (*M* = 4.67, *SD* = 0.91), *t*(419) = −5.38, *p* < .001. Older adults also reported higher PA levels than the younger group, as Table 1 shows.

**TABLE 1:** Comparison of variables between the sample of older and younger adults.

	Older adults (*n* = 241)		Younger adults (*n* = 180)		*t/χ* ^2^, *p*
	Mean/%	SD	Mean/%	SD	
Age	68.27	5.40	20.29	2.00	113.44, *p* < .001
Women	60%	–	63%	–	0.30, *p* = .59
BMI	22.97	2.87	20.70	2.97	7.90, *p* < .001
Chronic condition	47%	–	22%	–	27.94, *p* < .001
Perceived health (1–5)	3.35	0.67	3.05	0.73	4.41, *p* < .001
Education level (1–7)	4.28	1.55	4.82	0.94	−4.14, *p* < .001
Immediate benefit (0–7)	5.82	1.19	5.30	1.46	4.01, *p* < .001
Health consciousness (1–5)	4.04	0.55	3.65	0.56	7.15, *p* < .001
Future‐time perspective (1–7)	4.09	1.23	4.67	0.91	−5.38, *p* < .001
Baseline PA (MET‐min/week)	3630	2581	3028	2422	2.43, *p* = .015
Follow‐up PA (MET‐min/week)	3782	2459	2999	2634	2.89, *p* = .004

*Note*: *t*‐tests were used for continuous variables, and categorical variables were compared using chi‐square tests.

Abbreviation: PA, physical activity.

The moderated mediation model fitted the data reasonably well, Chi‐square = 12.08, *df* = 8, *p* = .15, CFI = .99, TLI = .97, RMSEA = .04 and SRMR = .02. Being in the older group was associated with higher health consciousness, *b* = 0.31 (95% bias‐corrected bootstrap CI: .19, .43), a lower FTP, *b* = −.26 (−.39, −.14) and a higher level of PA, *b* = .16 (.04, .28). The moderating effect of FTP on the relation between immediate benefits and physical activity was not significant, *b* = −.09 (−.18, .002). Health consciousness did not moderate the relation, b = .08 (−.02, .19). Although the moderating effects of FTP and health consciousness were not significant, the indirect effects they were involved in were found significant, as shown below.

There was an overall significant mediated moderation when both FTP and health consciousness were considered, *b* = .05 (.01, .10). When considered separately, the mediated moderation via FTP was significant, *b* = .02 (.002, .06), while the mediated moderation via health consciousness was not, *b* = .03 (−.01, .07). The results of the model are shown in Table 2 and Figure 1. The results of the simple slope analyses revealed that the relation between immediate benefits and PA was significant for those with a more limited FTP, *b* = .13 (.002, .26), but not significant for those with a more expansive FTP, *b* = −.05 (−.24, .12).

**TABLE 2:** Regression coefficients of the Mediated Moderation Model.

	HC	FTP	IB × HC	IB × FTP	MET
Direct effects					
Immediate benefit (IB)			−.15 (−.34, .03)	−.16 (−.34, .02)	.04 (−.09, .16)
Age group (AG)	.**31 (.19, .43)** ^**a**^	**−.26 (−.39, −.14)** ^**b**^			.**16 (.04, .28)**
Health consciousness (HC)					.10 (−.04, .23)
Future‐time perspective (FTP)					.10 (−.01, .20)
IB × AG			.**31 (.19, .43)** ^**a**^	**−.26 (−.39, −.14)** ^**b**^	**−.16 (−.27, −.05)**
IB × HC					.08 (−.02, .19)
IB × FTP					−.09 (−.18, .002)
Indirect effects					
Total indirect effect					.**05 (.01, .10)**
IB × AG → IB × HC →					.03 (−.01, .07)
IB × AG → IB × FTP →					.**02 (.002, .06)**

*Note*: The scores of MET‐minute/week were square root transformed to enhance distribution normality. In the brackets are the 95% bias‐corrected bootstrap confidence intervals. Significant coefficients are in boldface. Three decimal places are used for coefficients smaller than .01 to show better precision. ^a,b^The regression coefficients with the same superscript were constrained to be equal for correct model specification. Mediated moderation: 1. IB × AG → IB × FTP → PA: .02 (95%CI: .002, .06). 2. IB × AG → IB × HC → PA: .03 (95%CI: −.01, .07). Total mediated moderation: .05 (95%CI: .01, .10).

Abbreviation: MET, metabolic equivalent of task.

Unexpectedly, age group moderated the relation between immediate benefits and PA in a negative direction, *b* = −.16 (−.27, −.05). The results of simple slope analyses showed the immediate benefits were associated with PA among younger adults, *b* = .22 (.07, .37), but not among older adults, *b* = −.10 (−.29, .07).

The detailed results of the sensitivity analyses are presented in Tables S1 and S2. In both sets of sensitivity analyses, all the directions of the interested effects remained the same. Nonetheless, the magnitudes of the mediated moderation effects were slightly reduced and became non‐significant. In the discussion section, we interpreted the significant effects in the model without covariates. These results provided more confidence in statistical influence. We noted the necessary cautions for interpretation in the limitation section.

## DISCUSSION

The current study aimed to investigate whether increased age was associated with shortened FTP and increased health consciousness, which strengthened the association between the importance of immediate benefits and subsequent PA behaviour. We considered that the results marginally supported the mediated moderation effect of FTP, as the effect did not remain significant in the sensitivity analyses. As predicted, older adults perceived future time as more limited than younger adults. Subsequently, the age‐related reduction in FTP strengthened the association between immediate benefits and PA. However, independent of the mediated moderating effects, we observed a direct age moderation in an unexpected direction, that is the association between immediate benefits and subsequent PA was stronger among younger adults than older adults.

### Mediators of the age moderation

The results of the mediated moderation role of FTP were partially consistent with our expectations and echoed previous findings. We showed again that older adults perceived time as more limited than younger adults, as in previous studies (Li, 2019; Yeung et al., 2012). A limited FTP then strengthened the association between the importance of immediate benefits and subsequent PA. The results indicate that the matching between the dispositional FTP and the temporally oriented social cognition (i.e., the importance of immediate benefits of participating in PA) was effective in increasing PA. This extends the time perspective concordance hypothesis, which suggests that the matching between dispositional time perspective and contextual time perspective will yield favourable results (Li et al., 2021; Zimbardo & Boyd, 1999) from the contextual time perspective (e.g., temporally oriented interventions or classroom instructions, PA promotion messages focusing on future benefits) to temporally oriented social cognitions. That is, both external information and internal cognitions may contribute to the time perspective concordance.

Health consciousness increased with age in this study, as hypothesized, while the increased health consciousness did not strengthen the association between the importance of immediate benefits and subsequent PA. The non‐significant results may suggest that, contrary to our expectations, health consciousness does not align closely with immediate benefits to create the cognitive consistency needed to enhance the effects of immediate benefits. In other words, the immediate benefits of participating in physical activity may encompass many factors beyond health, or the trait of health consciousness may lack a clear temporal dimension. It is also possible that the results were due to insufficient statistical power, as they were close to reaching significance. Given that the effect size of this mediated moderation appears small, a future study with a larger sample size is necessary to draw a more definitive conclusion.

### Direct age moderation

An unexpected finding emerged in that the importance of immediate benefits was more predictive of the PA levels among younger adults than older adults, simultaneously with the mediated moderation effects being modelled in the analysis. We offer some potential interpretations below. Firstly, the unexpected age moderation indicates that there may be multiple mechanisms working in different directions. Some immediate benefits may be particularly motivating for younger adults. For instance, younger adults are inclined to exercise alone and for individual fitness goals, whereas older adults prefer to exercise with others (Steltenpohl et al., 2019). The immediate pleasure and satisfaction from exercising alone (e.g., feeling content after a good workout by oneself) may be a unique PA determinant for younger adults. In addition, younger adults' PA was motivated by autonomous regulation (i.e., intrinsic motivation) and introjected regulation (i.e., internalized external motivation) and discouraged by external regulations, whereas older adults' PA was mostly motivated by autonomous motivation (Brunet & Sabiston, 2011). It is possible that the immediate reliefs from external demands for PA (e.g., distracting oneself from some negative feelings, satisfying the expectation from significant others to stay fit) may also be unique for younger adults. We did not differentiate the types of immediate benefits in this study. Future studies may include different types of immediate benefits to better understand the mechanisms and explore other mediated moderations.

Secondly, older adults were more health‐conscious, as indicated by the current findings and might focus more on the immediate benefits in the health domain (e.g., being more energized, feeling less muscle tension and joint stiffness) than younger adults. This might result in high and homogenous scores in the importance of immediate benefits among older adults (*Mean* = 5.82 out of 7; *SD* = 1.19). This range restriction might limit the strength of the association between the importance of immediate benefits and subsequent PA in older adults.

### Limitations

First, no causation could be concluded from the current correlation data. Future studies can adopt experimental or more rigorous longitudinal designs. Second, the self‐report of PA might include recall and desirability biases. Objective measurements such as accelerometers are preferred in future studies. Third, the effects of interest were slightly reduced and became non‐significant when covariates were included, indicating the statistical power might be marginal for the current effect sizes. The covariates might also account for some of the mediated moderation effects. For instance, FTP and health consciousness were weakly correlated with perceived health, indicating a need for further exploration in future studies with greater statistical power. Hence, the results should be interpreted with caution. Fourth, the participants were relatively active and high‐functioning older adults and university students, which might limit their generalizability. Fifth, we did not consider the role of the long‐term benefits of PA in the FTP‐PA relationship. Incorporating it in future studies would help us reveal the complex interplay between FTP, health consciousness and PA. Finally, the item on immediate benefits could not reflect the different domains of benefits, such as health, social and psychological benefits, which limited our ability to understand the underlying mechanisms. Besides, the high scores in older adults might reflect a potential ceiling effect.

## CONCLUSION

This study was one of the first to investigate age differences and their temporal mechanisms in the motivational effects of immediate benefits on PA behaviour. Mediated moderation models have been advocated for unpacking moderation effects in cross‐cultural studies (Ng et al., 2019). Our results demonstrate that these models are also effective in unpacking the mechanisms of age moderation. In addition, several insights derived from this study include obtaining further evidence for and potential expansion of the time perspective concordance hypothesis (Li et al., 2021), suggesting the need for more understanding of why immediate benefits motivate younger adults to participate in PA and questioning whether health consciousness can be present‐oriented. Replications are needed to further support the unexpected and marginal findings. The speculative mechanisms can also inform future investigations. Experimental studies should be conducted to establish the causal relations in the future. Time‐based strategies can be included in future intervention studies to promote PA.

## AUTHOR CONTRIBUTIONS

**Kin‐Kit Li:** Conceptualization; methodology; formal analysis; funding acquisition; writing – original draft. **Wanying Zhao:** Investigation; project administration; writing – review and editing. **Cyrus Lap Kwan Leung:** Methodology; data curation; writing – review and editing.

## CONFLICT OF INTEREST STATEMENT

None declared.

## Supporting information


Data S1:

## Data Availability

The data supporting this study's findings are available on request from the corresponding authors.

## References

[bjhp70006-bib-0001] Adams, J. (2009). The mediating role of time perspective in socio‐economic inequalities in smoking and physical activity in older English adults. Journal of Health Psychology, 14, 794–799. 10.1177/1359105309338979 19687116

[bjhp70006-bib-0002] Allen, M. S. , Iliescu, D. , & Greiff, S. (2022). Single item measures in psychological science. European Journal of Psychological Assessment, 38, 1–5. 10.1027/1015-5759/a000699

[bjhp70006-bib-0003] Becker, C. M. , & Arnold, W. (2004). Health promoting behaviors of older Americans versus young and middle aged adults. Educational Gerontology, 30, 835–844. 10.1080/03601270490507277

[bjhp70006-bib-0004] Brunet, J. , & Sabiston, C. M. (2011). Exploring motivation for physical activity across the adult lifespan. Psychology of Sport and Exercise, 12(2), 99–105. 10.1016/j.psychsport.2010.09.006

[bjhp70006-bib-0005] Bull, F. C. , Al‐Ansari, S. S. , Biddle, S. , Borodulin, K. , Buman, M. P. , Cardon, G. , Carty, C. , Chaput, J.‐P. , Chastin, S. , Chou, R. , Dempsey, P. C. , DiPietro, L. , Ekelund, U. , Firth, J. , Friedenreich, C. M. , Garcia, L. , Gichu, M. , Jago, R. , Katzmarzyk, P. T. , … Willumsen, J. F. (2020). World Health Organization 2020 guidelines on physical activity and sedentary behaviour. British Journal of Sports Medicine, 54(24), 1451–1462. 10.1136/bjsports-2020-102955 33239350 PMC7719906

[bjhp70006-bib-0006] Campbell, P. G. , MacAuley, D. , McCrum, E. , & Evans, A. (2001). Age differences in the motivating factors for exercise. Journal of Sport and Exercise Psychology, 23, 191–199. 10.1123/jsep.23.3.191

[bjhp70006-bib-0007] Carstensen, L. L. , Fung, H. H. , & Charles, S. T. (2003). Socioemotional selectivity theory and the regulation of emotion in the second half of life. Motivation and Emotion, 27(2), 103–123. 10.1023/A:1024569803230

[bjhp70006-bib-0008] Carstensen, L. L. , & Lang, F. R. (1996). Future Time Perspective Scale. 10.1037/t31314-000

[bjhp70006-bib-0009] Daugherty, J. R. , & Brase, G. L. (2010). Taking time to be healthy: Predicting health behaviors with delay discounting and time perspective. Personality and Individual Differences, 48(2), 202–207. 10.1016/j.paid.2009.10.007

[bjhp70006-bib-0010] Devereux‐Fitzgerald, A. , Powell, R. , Dewhurst, A. , & French, D. P. (2016). The acceptability of physical activity interventions to older adults: A systematic review and meta‐synthesis. Social Science & Medicine, 158, 14–23. 10.1016/j.socscimed.2016.04.006 27104307

[bjhp70006-bib-0011] Evans, M. B. , Shanahan, E. , Leith, S. , Litvak, N. , & Wilson, A. E. (2019). Living for today or tomorrow? Self‐regulation amidst proximal or distal exercise outcomes. Applied Psychology. Health and Well‐Being, 11(2), 304–327. 10.1111/aphw.12160 30912301

[bjhp70006-bib-0012] Faul, F. , Erdfelder, E. , Buchner, A. , & Lang, A.‐G. (2009). Statistical power analyses using G*power 3.1: Tests for correlation and regression analyses. Behavior Research Methods, 41(4), 1149–1160. 10.3758/BRM.41.4.1149 19897823

[bjhp70006-bib-0013] Festinger, L. (1957). A theory of cognitive dissonance. Stanford University Press.

[bjhp70006-bib-0014] Gawronski, B. (2012). Back to the future of dissonance theory: Cognitive consistency as a Core motive. Social Cognition, 30(6), 652–668. 10.1521/soco.2012.30.6.652

[bjhp70006-bib-0015] Gawronski, B. , & Brannon, S. M. (2019). What is cognitive consistency, and why does it matter? In B. Gawronski & S. M. Brannon (Eds.), Cognitive dissonance: Reexamining a pivotal theory in psychology (2nd ed., pp. 91–116). American Psychological Association. 10.1037/0000135-005

[bjhp70006-bib-0016] Green, L. , Fry, A. F. , & Myerson, J. (1994). Discounting of delayed rewards: A life‐span comparison. Psychological Science, 5(1), 33–36. 10.1111/j.1467-9280.1994.tb00610.x

[bjhp70006-bib-0017] Guthrie, L. C. , Butler, S. C. , Lessl, K. , Ochi, O. , & Ward, M. M. (2014). Time perspective and exercise, obesity, and smoking: Moderation of associations by age. American Journal of Health Promotion, 29(1), 9–16. 10.4278/ajhp.130122-QUAN-39 24200252 PMC4183963

[bjhp70006-bib-0018] Hall, P. A. , Fong, G. T. , & Sansone, G. (2015). Time perspective as a predictor of healthy behaviors and disease‐mediating states. In M. Stolarski , N. Fieulaine , & W. van Beek (Eds.), Time perspective theory; review, research and application: Essays in honor of Philip G. Zimbardo (pp. 339–352). Springer International Publishing. 10.1007/978-3-319-07368-2_22

[bjhp70006-bib-0019] Hamilton, J. M. , Kives, K. D. , Micevski, V. , & Grace, S. L. (2003). Time perspective and health‐promoting behavior in a cardiac rehabilitation population. Behavioral Medicine, 28(4), 132–139. 10.1080/08964280309596051 14663920

[bjhp70006-bib-0020] Harmon‐Jones, E. , Amodio, D. M. , & Harmon‐Jones, M. (2009). Action‐based model of dissonance: A review, integration, and expansion of conceptions of cognitive conflict. Advances in Experimental Social Psychology, 41, 119–166. 10.1016/S0065-2601(08)00403-6

[bjhp70006-bib-0021] Hong, H. (2009). Scale development for measuring health consciousness: Re‐conceptualization. *Research that matters to the practice*. Holiday Inn University of Miami, Coral Gables, Florida.

[bjhp70006-bib-0022] Hooker, K. (1992). Possible selves and perceived health in older adults and college students. Journal of Gerontology, 47(2), P85–P95. 10.1093/geronj/47.2.P85 1538073

[bjhp70006-bib-0023] John, D. , & Lang, F. R. (2015). Subjective acceleration of time experience in everyday life across adulthood. Developmental Psychology, 51(12), 1824–1839. 10.1037/dev0000059 26414094

[bjhp70006-bib-0024] Kwan, J. L. Y. , & Chan, W. (2018). Variable system: An alternative approach for the analysis of mediated moderation. Psychological Methods, 23(2), 262–277. 10.1037/met0000160 29172615

[bjhp70006-bib-0025] Lang, F. R. , & Carstensen, L. L. (2002). Time counts: Future time perspective, goals, and social relationships. Psychology and Aging, 17(1), 125–139. 10.1037/0882-7974.17.1.125 11931281

[bjhp70006-bib-0026] Leigh, J. P. , Hubert, H. B. , & Romano, P. S. (2005). Lifestyle risk factors predict healthcare costs in an aging cohort. American Journal of Preventive Medicine, 29(5), 379–387. 10.1016/j.amepre.2005.08.005 16376700

[bjhp70006-bib-0027] Lens, W. , Paixão, M. P. , Herrera, D. , & Grobler, A. (2012). Future time perspective as a motivational variable: Content and extension of future goals affect the quantity and quality of motivation. Japanese Psychological Research, 54(3), 321–333. 10.1111/j.1468-5884.2012.00520.x

[bjhp70006-bib-0028] Li, K.‐K. (2013). Domain dimensionality and temporality of outcome expectancy for physical activity among middle‐aged and older Chinese adults: A latent profile analysis. Psychology of Sport and Exercise, 14(5), 682–691. 10.1016/j.psychsport.2013.05.007

[bjhp70006-bib-0029] Li, K.‐K. (2019). Negotiations between health and social goals over the lifespan: The role of future time perspective. Journal of Health Psychology, 24(9), 1233–1244. 10.1177/1359105317693911 28810413

[bjhp70006-bib-0030] Li, K.‐K. , Cheng, S.‐T. , & Fung, H. H. (2014). Effects of message framing on self‐report and accelerometer‐assessed physical activity across age and gender groups. Journal of Sport and Exercise Psychology, 36, 40–51. 10.1123/jsep.2012-0278 24501143

[bjhp70006-bib-0031] Li, K.‐K. , Yip, H. Y. V. , & Wong, Y. S. N. (2021). Effects of dispositional and instructional time perspective on academic performance and motivations among primary school students: A concordance hypothesis. Frontiers in Education, 6, 771740. 10.3389/feduc.2021.771740

[bjhp70006-bib-0032] Löckenhoff, C. E. , & Carstensen, L. L. (2007). Aging, emotion, and health‐related decision strategies: Motivational manipulations can reduce age differences. Psychology and Aging, 22(1), 134–146. 10.1037/0882-7974.22.1.134 17385990

[bjhp70006-bib-0033] Macfarlane, D. J. , Lee, C. C. Y. , Ho, E. Y. K. , Chan, K. L. , & Chan, D. T. S. (2007). Reliability and validity of the Chinese version of IPAQ (short, last 7 days). Journal of Science and Medicine in Sport, 10(1), 45–51. 10.1016/j.jsams.2006.05.003 16807105

[bjhp70006-bib-0034] Muthén, L. K. , & Muthén, B. O. (1998). Mplus User's Guide (Eighth ed.). Muthén & Muthén.

[bjhp70006-bib-0035] Netz, Y. , & Raviv, S. (2004). Age differences in motivational orientation toward physical activity: An application of social‐cognitive theory. The Journal of Psychology, 138(1), 35–48. 10.3200/JRLP.138.1.35-48 15098713

[bjhp70006-bib-0036] Ng, J. C. K. , Chan, W. , Kwan, J. L. Y. , & Chen, S. X. (2019). Unpacking structure‐oriented cultural differences through a mediated moderation model: A tutorial with an empirical illustration. Journal of Cross‐Cultural Psychology, 50(3), 358–380. 10.1177/0022022118821183

[bjhp70006-bib-0037] Noordewier, M. K. , Topolinski, S. , & Van Dijk, E. (2016). The temporal dynamics of surprise. Social and Personality Psychology Compass, 10(3), 136–149. 10.1111/spc3.12242

[bjhp70006-bib-0038] Orbell, S. , & Kyriakaki, M. (2008). Temporal framing and persuasion to adopt preventive health behavior: Moderating effects of individual differences in consideration of future consequences on sunscreen use. Health Psychology, 27(6), 770–779. 10.1037/0278-6133.27.6.770 19025273

[bjhp70006-bib-0039] Reiner, M. , Niermann, C. , Jekauc, D. , & Woll, A. (2013). Long‐term health benefits of physical activity – A systematic review of longitudinal studies. BMC Public Health, 13(1), 813. 10.1186/1471-2458-13-813 24010994 PMC3847225

[bjhp70006-bib-0040] Seaman, K. L. , Gorlick, M. A. , Vekaria, K. M. , Hsu, M. , Zald, D. H. , & Samanez‐Larkin, G. R. (2016). Adult age differences in decision making across domains: Increased discounting of social and health‐related rewards. Psychology and Aging, 31(7), 737–746. 10.1037/pag0000131 27831713 PMC5127408

[bjhp70006-bib-0041] Stahl, S. T. , & Patrick, J. H. (2012). Adults' future time perspective predicts engagement in physical activity. The Journals of Gerontology. Series B, Psychological Sciences and Social Sciences, 67(4), 413–416. 10.1093/geronb/gbr118 22042760

[bjhp70006-bib-0042] Steltenpohl, C. N. , Shuster, M. , Peist, E. , Pham, A. , & Mikels, J. A. (2019). Me time, or we time? Age differences in motivation for exercise. The Gerontologist, 59(4), 709–717. 10.1093/geront/gny038 29688424 PMC6630158

[bjhp70006-bib-0043] Strathman, A. , Gleicher, F. , Boninger, D. S. , & Edwards, C. S. (1994). The consideration of future consequences: Weighing immediate and distant outcomes of behavior. Journal of Personality and Social Psychology, 66(4), 742–752. 10.1037/0022-3514.66.4.742

[bjhp70006-bib-0044] Strunk, B. C. , Ginsburg, P. B. , & Banker, M. I. (2006). The effect of population aging on future hospital demand. Health Affairs, 25(Supplement 1), W141–W149. 10.1377/hlthaff.25.w141 16569646

[bjhp70006-bib-0045] United Nations Department for Economic and Social Affairs . (2023). World Population Prospects 2022: Summary of Results. United Nations Fund for Population Activities.

[bjhp70006-bib-0046] Wanous, J. P. , Reichers, A. E. , & Hudy, M. J. (1997). Overall job satisfaction: How good are single‐item measures? Journal of Applied Psychology, 82(2), 247–252. 10.1037/0021-9010.82.2.247 9109282

[bjhp70006-bib-0047] Warburton, D. E. , Nicol, C. W. , & Bredin, S. S. (2006). Health benefits of physical activity: The evidence. CMAJ, 174(6), 801–809. 10.1503/cmaj.051351 16534088 PMC1402378

[bjhp70006-bib-0048] Whelan, R. , & McHugh, L. A. (2009). Temporal discounting of hypothetical monetary rewards by adolescents, adults, and older adults. The Psychological Record, 59(2), 247–258. 10.1007/BF03395661

[bjhp70006-bib-0049] Williamson, C. , Baker, G. , Mutrie, N. , Niven, A. , & Kelly, P. (2020). Get the message? A scoping review of physical activity messaging. International Journal of Behavioral Nutrition and Physical Activity, 17(1), 51. 10.1186/s12966-020-00954-3 32295613 PMC7160981

[bjhp70006-bib-0050] Wininger, S. R. , & Desena, T. M. (2012). Comparison of future time perspective and self‐determination theory for explaining exercise behavior. Journal of Applied Biobehavioral Research, 17(2), 109–128. 10.1111/j.1751-9861.2012.00081.x

[bjhp70006-bib-0051] Woolley, K. , & Fishbach, A. (2017). When intrinsic motivation and immediate rewards overlap. In Motivation‐cognition interface (pp. 1–19). Routledge.

[bjhp70006-bib-0052] World Health Organization . (2022). Global status report on physical activity 2022: Country profiles. World Health Organization.

[bjhp70006-bib-0053] Wrosch, C. , Scheier, M. F. , & Miller, G. E. (2013). Goal adjustment capacities, subjective well‐being, and physical health. Social and Personality Psychology Compass, 7(12), 847–860. 10.1111/spc3.12074 25177358 PMC4145404

[bjhp70006-bib-0054] Yeung, D. Y. , Fung, H. H. , & Kam, C. (2012). Age differences in problem solving strategies: The mediating role of future time perspective. Personality and Individual Differences, 53(1), 38–43. 10.1016/j.paid.2012.02.014

[bjhp70006-bib-0055] Ziegelmann, J. P. , Lippke, S. , & Schwarzer, R. (2006). Subjective residual life expectancy in health self‐regulation. The Journals of Gerontology Series B: Psychological Sciences and Social Sciences, 61(4), P195–P201. 10.1093/geronb/61.4.P195 16855031

[bjhp70006-bib-0056] Zimbardo, P. G. , & Boyd, J. N. (1999). Putting time in perspective: A valid, reliable individual‐differences metric. Journal of Personality and Social Psychology, 77(6), 1271–1288. 10.1037/0022-3514.77.6.1271

