# Supplementary material for: Explaining the age‐moderation effects in the relation between immediate benefits and physical activity: A mediated moderation analysis
Source: Br J Health Psychol. 2025 Jul 14;30(3):e70006. doi: 10.1111/bjhp.70006 (PMC12260479; doi:10.1111/bjhp.70006)
Supplement: Supplementary file 1 — Data S1: [file BJHP-30-0-s001.docx]

**Supplementary online materials**

Article title:

Explaining the age-moderation effects in the relation between immediate benefits and physical activity: A mediated moderation analysis

**Table S1**

*Regression coefficients of the Mediated Moderation Model with 5 covariates included*

|  | HC | FTP | IB × HC | IB × FTP | Time 2 MET |
| --- | --- | --- | --- | --- | --- |
| Direct effects |  |  |  |  |  |
| Women | -.06 (-.15, .04) | -.07 (-.15, .03) | -.007 (-.11, .09) | **.11 (.02, .21)** | -.10 (-.21, .004) |
| BMI | -.06 (-.16, .03) | .04 (-.02, .14) | .**09 (.008, .19)** | .009 (-.09, .11) | .10 (-.001, .20) |
| Chronic condition | .000 (-.09, .09) | .001 (-.09, .10) | -.04 (-.14, .07) | -.03 (-.14, .08) | .03 (-.07, .14) |
| Perceived health | **.16 (.06, .26)** | **.40 (.32, .50)** | .03 (-.09, .16) | .07 (-.05, .20) | .09 (-.03, .21) |
| Education level | -.09 (-.19, .007) | -.04 (-.13, .05) | .03 (-.07, .13) | .04 (-.05, .14) | -.06 (-.17, .06) |
| Immediate benefit (IB) |  |  | -.15 (-.36, .03) | -.18 (-.36, .008) | .04 (-.09, .17) |
| Age group (AG) | **.29 (.17, .42)^a^** | **-.33 (-.46, -.21)^b^** |  |  | .07 (-.07, .21) |
| Health consciousness (HC) |  |  |  |  | .11 (-.04, .24) |
| Future time perspective (FTP) |  |  |  |  | .06 (-.07, .17) |
| IB × AG |  |  | **.29 (.17, .42)^a^** | **-.33 (-.46, -.21)^b^** | **-.14 (-.25, -.03)** |
| IB × HC |  |  |  |  | .08 (-.03, .19) |
| IB × FTP |  |  |  |  | -.07 (-.16, .03) |
|  |  |  |  |  |  |
| Indirect effects |  |  |  |  |  |
| Total indirect effect |  |  |  |  | .04 (-.002, .10) |
| IB × AG 🡪 IB × HC 🡪 |  |  |  |  | .02 (-.006, .07) |
| IB × AG 🡪 IB × FTP 🡪 |  |  |  |  | .02 (-.006, .06) |

Note. MET = metabolic equivalent of task. The scores of T2 MET-minute/week were square root transformed to enhance distribution normality. In the brackets are the 95% bias-corrected bootstrapped confidence intervals. Significant coefficients are in boldface. Three decimal places are used for coefficients smaller than .01 to show better precision.

^a,b^ The regression coefficients with the same superscript were constrained to be equal for correct model specification.

**Table S2**

*Regression coefficients of the Mediated Moderation Model with 6 covariates included*

|  | HC | FTP | IB × HC | IB × FTP | Time 2 MET |
| --- | --- | --- | --- | --- | --- |
| Direct effects |  |  |  |  |  |
| Women | -.02 (-.11, .07) | -.05 (-.14, .04) | -.008 (-.12, .09) | **.11 (.01, .21)** | -.04 (-.13, .05) |
| BMI | -.08 (-.17, .02) | .04 (-.05, .13) | **.10 (.007, .19)** | .01 (-.09, .11) | .05 (-.04, .12) |
| Chronic condition | -.009 (-.10, .08) | -.003 (-.10, .09) | -.04 (-.14, .07) | -.03 (-.14, .08) | .009 (-.08, .10) |
| Perceived health | **.12 (.02, .21)** | **.39 (.30, .48)** | .03 (-.09, .16) | .08 (-.05, .20) | -.006 (-.10, .08) |
| Education level | -.08 (-.17, .02) | -.03 (-.12, .06) | .03 (-.07, .13) | .04 (-.05, .13) | -.02 (-.12, .07) |
| Baseline PA (MET-min/wk) | **.21 (.12, .30)** | .09 (-.001, .18) | -.01 (-.11, .09) | -.01 (-.12, .09) | **.57 (.45, .68)** |
| Immediate benefit (IB) |  |  | -.15 (-.36, .03) | -.18 (-.36, .006) | -.006 (-.11, .10) |
| Age group (AG) | **.29 (.17, .42)^a^** | **-.33 (-.47, -.21)^b^** |  |  | .11 (-.01, .22) |
| Health consciousness (HC) |  |  |  |  | -.01 (-.13, .10) |
| Future time perspective (FTP) |  |  |  |  | .04 (-.07, .14) |
| IB × AG |  |  | **.29 (.17, .42)^a^** | **-.33 (-.47, -.21)^b^** | **-.12 (-.21, -.03)** |
| IB × HC |  |  |  |  | .06 (-.02, .15) |
| IB × FTP |  |  |  |  | -.05 (-.13, .03) |
|  |  |  |  |  |  |
| Indirect effects |  |  |  |  |  |
| Total indirect effect |  |  |  |  | .03 (-.007, .08) |
| IB × AG 🡪 IB × HC 🡪 |  |  |  |  | .02 (-.005, .05) |
| IB × AG 🡪 IB × FTP 🡪 |  |  |  |  | .02 (-.01, .05) |

Note. PA = physical activity; MET = metabolic equivalent of task. The scores of T2 MET-minute/week were square root transformed to enhance distribution normality. In the brackets are the 95% bias-corrected bootstrapped confidence intervals. Significant coefficients are in boldface. Three decimal places are used for coefficients smaller than .01 to show better precision.

^a,b^ The regression coefficients with the same superscript were constrained to be equal for correct model specification.
